# Evaluation of Machine Learning Models for Proteoform Retention and Migration Time Prediction in Top-Down Mass Spectrometry

**DOI:** 10.1021/acs.jproteome.2c00124

**Published:** 2022-05-26

**Authors:** Wenrong Chen, Elijah N. McCool, Liangliang Sun, Yong Zang, Xia Ning, Xiaowen Liu

**Affiliations:** † Department of BioHealth Informatics, Indiana University-Purdue University Indianapolis, Indianapolis, Indiana 46202, United Staes; ‡ Department of Chemistry, Michigan State University, East Lansing, Michigan 48824, United Staes; § Department of Biostatics and Health Data Sciences, Indiana University School of Medicine, Indianapolis, Indiana 46202, United Staes; ∥ Department of Biomedical Informatics, The Ohio State University, Columbus, Ohio 43210, United Staes; ⊥ Department of Computer Science and Engineering, The Ohio State University, Columbus, Ohio 43210, United Staes; # Translational Data Analytics Institute, The Ohio State University, Columbus, Ohio 43210, United Staes; ¶ Tulane Center for Biomedical Informatics and Genomics, Tulane University, New Orleans, Louisiana 70112, United Staes; ∇ Deming Department of Medicine, Tulane University, New Orleans, Louisiana 70112, United Staes

**Keywords:** top-down mass spectrometry, retention/migration time prediction, machine learning

## Abstract

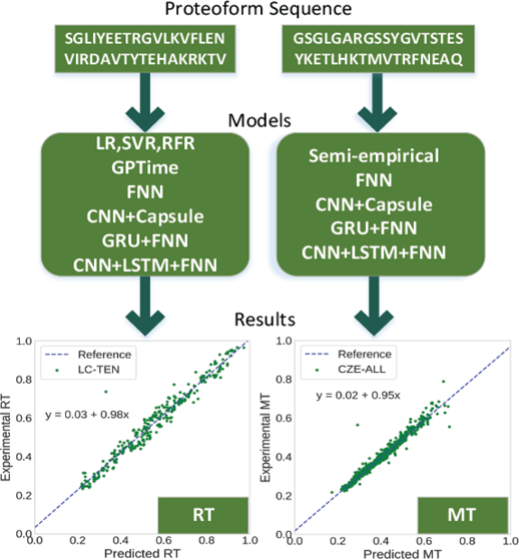

Reversed-phase liquid chromatography (RPLC) and capillary zone electrophoresis (CZE) are two primary proteoform separation methods in mass spectrometry (MS)-based top-down proteomics. Proteoform retention time (RT) prediction in RPLC and migration time (MT) prediction in CZE provide additional information for accurate proteoform identification and quantification. While existing methods are mainly focused on peptide RT and MT prediction in bottom-up MS, there is still a lack of methods for proteoform RT and MT prediction in top-down MS. We systematically evaluated eight machine learning models and a transfer learning method for proteoform RT prediction and five models and the transfer learning method for proteoform MT prediction. Experimental results showed that a gated recurrent unit (GRU)-based model with transfer learning achieved a high accuracy (*R* = 0.978) for proteoform RT prediction and that the GRU-based model and a fully connected neural network model obtained a high accuracy of *R* = 0.982 and 0.981 for proteoform MT prediction, respectively.

## 1 Introduction

Top-down mass spectrometry (MS), an important complementary method for bottom-up MS, has been widely used in proteoform identification, characterization, and quantification.^1−3^ The main difference between the two approaches is that top-down MS analyzes long intact proteoforms, while bottom-up MS studies short peptides resulting from proteoform proteolytic digestion. Top-down MS enables researchers to study complex proteoforms and post-translational modification (PTM) patterns in proteoforms^4^ owing to its ability to identify whole proteoforms.

Many proteoform separation techniques have been used to increase proteoform coverage in top-down MS,^5,6^ which is desirable in proteoform-wide studies for proteoform function analysis and proteoform biomarker discovery.^7^ Liquid chromatography (LC) and capillary zone electrophoresis (CZE) are two main techniques for proteoform separation in top-down proteomics.^7,8^ In an LC experiment, proteoforms are separated based on their hydrophobicity, size, or other properties using an LC column. There are many LC methods, such as reversed-phase liquid chromatography (RPLC),^9^ size exclusion chromatography (SEC),^10^ and ion exchange chromatography (IEC).^11^ RPLC is one of the most used methods owing to its high separation performance in top-down MS.^12,13^ In CZE-based separation, proteoforms are injected into a capillary filled with a background electrolyte on which an electric field is applied. Proteoforms with different charges and hydrodynamic radii are separated based on their electrophoretic mobility.^14^ Many studies showed that CZE is a highly efficient method for proteoform separation, achieving over a million theoretical plates for some samples.^15−17^

Predicting proteoform retention times (RTs) in RPLC–MS and migration times (MTs) in CZE-MS can increase the accuracy of proteoform identification in top-down tandem mass spectrometry (MS/MS). An incorrect proteoform identified by top-down MS/MS tends to have a large difference between its empirical and theoretical RTs or MTs. Accurate prediction of proteoform RTs or MTs allows for increasing proteoform identification accuracy by filtering out identifications with inconsistent theoretical and empirical RTs or MTs.

Many methods have been proposed for RT prediction in bottom-up MS,^18^ which can be divided into three categories: library-based methods, index-based methods, and machine learning-based methods. In library-based methods,^19^ a library is built and maintained for peptides with known RTs identified from previous LC experiments, and peptide RTs are predicted using the library. In index-based methods, retention coefficients of amino acids are first computed using experimental data, and the RT of a peptide is predicted based on the sum of the retention coefficients of its amino acids. For example, SSRCalc^20,21^ produced a high accuracy in RT prediction using retention coefficients.

Machine learning-based methods achieved the best performance for RT prediction in bottom-up MS. Quantitative structure-retention relationship (QSRR)^22^ calculates and selects significant chemical descriptors of peptides and uses a regression method to predict RTs. RTPredict^23,24^ and ELUDE^25^ extract discriminant features of amino acids in peptides and predict RTs using support vector machines. GPTime^26^ utilizes the features from ELUDE and a Gaussian process regression model^27^ to obtain a high accuracy for RT prediction. Recently, many deep learning models have been reported for peptide RT prediction in bottom-up MS,^28,29^ which can be divided into three groups: convolutional neural network (CNN)-based models, such as DeepRT+^30^ and DeepLC,^31^ recurrent neural network-based models, such as Prosit^32^ and DeepMass,^33^ and hybrid models with both convolutional and recurrent layers, such as DeepDIA^34^ and AutoRT.^35^ Specifically, DeepRT+ uses convolutional and capsule layers;^36^ Prosit employs gated recurrent units (GRUs), an attention layer, and fully connected layers; and DeepDIA combines convolutional, long short-term memory (LSTM), and fully connected layers. These deep learning models significantly increased the accuracy of peptide RT prediction (Table S1). For CZE MT prediction, the peptide size and charge are two major features that affect peptide electrophoretic mobilities and MTs.^14,37−40^ A classical semi-empirical model based on the two features produced an accuracy of *R*^2^ = 0.974 for peptide electrophoretic mobility prediction on a bottom-up CZE-MS yeast data set.^14^ After model optimization, the accuracy was improved to *R*^2^ = 0.991.

The RT and MT prediction problems in top-down MS share a high similarity with those in bottom-up MS, and the main difference is that proteoforms in top-down MS are longer than peptides in bottom-up MS. While many methods have been proposed for peptide RT/MT prediction, only several studies have been done for proteoform RT/MT prediction. The main reason is that high-quality training data sets are lacking for proteoform RT and MT prediction. Chen et al.^41^ extended the semi-empirical model^14^ for peptide MT prediction to proteoform MT prediction and obtained an *R*^2^ = 0.98 on an *Escherichia coli* CZE-MS data set. To the best of our knowledge, there have been no studies of the RT prediction problem in top-down LC–MS.

In this article, we benchmarked the performance of eight machine learning models for proteoform RT prediction and five models for proteoform MT prediction. The models for proteoform RT prediction are logistic regression (LR), random Forest regression (RFR), support vector regression (SVR), GPTime,^26^ a fully connected neural network (FNN) model, and the GRU + FNN model in Prosit,^32^ the CNN + capsule model in DeepRT+,^30^ and the CNN + LSTM + FNN model in DeepDIA.^34^ The models for proteoform MT prediction are the semi-empirical model in the study of Chen et al.^41^ and the four neural network models. We also assessed a transfer learning method in which peptides are first employed for model pretraining, and then, proteoforms are used for model retraining. The method improved the prediction accuracy for some neural network models when the size of top-down MS training data was limited. Experimental results showed that the GRU + FNN model with transfer learning achieved a high accuracy for RT prediction (*R* = 0.978) and that the GRU + FNN and FNN models obtained a high accuracy for MT prediction (GRU + FNN: *R* = 0.982; FNN: *R* = 0.981).

## 2 Methods

### 2.1 Top-Down MS Data Sets

Two top-down MS/MS data sets were used in this study: one public RPLC–MS/MS data^42^ (MASSIVE: MSV000080257) and one CZE-MS/MS data.^6^ The RPLC–MS/MS data set was generated from ovarian tumor samples. A solid-phase extraction column (360 μm o.d. × 150 μm i.d.) was used for trapping and desalting before separation. The separation process was performed using a dual-pump Waters nanoACQUITY UPLC system (Milford, Massachusetts) and a 50 cm length analytical column (360 μm o.d. × 100 μm i.d.) packed with 3 μm diameter C2 (Separation Methods Technology, Newark, Delaware). 5 μL of the sample was loaded and separated with a 180 min gradient from 99% solvent A to 35% solvent A with a 0.3 μL/min flow rate (A: 0.2% formic acid in water, B: 0.2% formic acid in acetonitrile). The separation system was coupled with an Orbitrap Elite mass spectrometer (Thermo Fisher, San Jose, California). MS1 and MS/MS spectra were collected at a resolution of 240,000 and 120,000 at 200 *m*/*z*, respectively. The top four precursor ions in each MS1 spectrum were isolated with a 4 *m*/*z* window and fragmented with collision-induced dissociation (CID) at a normalized collision energy of 35%. Ten technical replicates were generated for the same sample.

The CZE-MS/MS data sets were obtained from SW480 and SW620 colon cancer cells.^6^ Sample proteins were first separated by an SEC column into six fractions, and then each fraction was injected into a linear polyacrylamide-coated fused silica capillary (1 m, 50 μm i.d., 360 μm o.d.) with 5% acetic acid as the background electrolyte. The electrospray voltage was 2–2.3 kV, and the separation voltage was 30 kV for 100 min. The CZE system was coupled with a Q-Exactive HF mass spectrometer (Thermo Fisher, San Jose, California). MS1 and MS/MS spectra were collected at a resolution of 120,000 at 200 *m*/*z*. The top five precursor ions in each MS1 spectrum were analyzed using HCD MS/MS. Three technical replicates were obtained for SW480 and SW620 cells with a total of 18 runs (6 fractions × 3 replicates) for SW480 cells and 18 runs (6 fractions × 3 replicates) for SW620 cells.

### 2.2 Proteoform Identification

All raw MS files were converted to centroided mzML files using msconvert in ProteoWizard.^43^ TopFD (version 1.4.0)^44^ was employed to deconvolute the centroided mass spectra to neutral monoisotopic masses of precursor and fragment ions. The deconvoluted MS/MS spectra were searched against the corresponding UniProt proteome database (version Octember 23, 2019) for proteoform identification using TopPIC (version 1.4.0).^44^ In database search, the error tolerance for precursor and fragment masses was set to 15 ppm, and unknown mass shifts were not allowed. Cysteine carbamidomethylation was specified as a fixed modification for the colon cancer cell data set, and no fixed modifications were set for the ovarian tumor data set. Proteoform-spectrum matches (PrSMs) reported by the database search were filtered with a stringent *E*-value cutoff of 10^–5^ to remove low confidence ones. These PrSMs were further clustered by merging PrSMs into the same cluster if the proteoforms of the PrSMs were from the same protein and the difference of their precursor masses was <2.2 Da. The PrSM with the best *E*-value in each cluster was reported, and PrSMs with N-terminal acetylation were filtered out. Details of the parameter settings of TopPIC are given in Table S2 in the Supporting Information. The apex RT/MT of a proteoform reported by TopFD was used as the empirical RT/MT of the proteoform and was further normalized by dividing it by the separation time of the experiment.

To combine proteoforms identified from multiple MS files of different samples, we grouped proteoforms into the same cluster if they were from the same protein and the difference of their molecular masses was <2.2 Da. In each cluster, the proteoform with the best PrSM (lowest *E*-value) was reported.

### 2.3 Machine Learning Models

A total of eight machine learning models were assessed for predicting proteoform RTs in top-down RPLC–MS: LR, RFR, SVR, the model in GPTime,^26^ an FNN model, the CNN + capsule model in DeepRT+,^30^ the GRU + FNN model in Prosit,^32^ and the CNN + LSTM + FNN model in DeepDIA.^34^ The last four models and the semi-empirical model in the study by Chen et al.^41^ were also benchmarked for predicting proteoform MTs in top-down CZE-MS. All the models were implemented in Python (version 3.6.8). The FNN and CNN + capsule models were implemented using the PyTorch package (version 1.18.1)^45^ and the GRU + FNN and CNN + LSTM + FNN models using the Keras package (version 2.1.1)^46^ with the TensorFlow backend (version 1.14.0). The machine learning models were trained on a computer with an Intel Xeon 2.20 GHz 10 core CPU, 192 GB memory, and an Nvidia Geforce Titan Xp GPU running the Ubuntu 18.04 operating system.

#### 2.3.1 GPTime Model for RT Prediction

The model in GPTime with 62 features^25,26^ was used for proteoform RT prediction in top-down MS. The first feature was the proteoform length, and the second was the sum of the bulkiness indexes^47^ of all amino acid residues in the proteoform. The other 60 features were computed for the 20 standard amino acids, each represented by three features: its hydrophobicity index,^48^ the number of occurrences, and the retention index. The retention indexes were obtained by training a linear regression model using experimental data.^25^ Gaussian process regression with the radial basis function kernel was used for proteoform RT prediction.^27^

#### 2.3.2 Semi-Empirical Model for MT Prediction

The semi-empirical model in the study of Chen et al.^41^ was adopted to predict proteoform MTs in CZE-MS, in which the MT of a proteoform is determined using two features: its molecular mass *M* and charge *Z*. The molecular mass is used to predict the size of the proteoform. The charge is estimated as the total number of positively charged amino acid residues (R, H, K, and the N-terminus) in the proteoform.^14^ The electrophoretic mobility of the proteoform is predicted as , where *a* and *b* are two parameters related to CZE settings.^41^ The electrophoretic mobility can be converted to its corresponding MT using
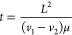
1where *L* is the capillary length, *v*_1_ is the CZE separation voltage, and *v*_2_ is the electrospray voltage in the experiment.

#### 2.3.3 Neural Network Models

An FNN model was built to predict proteoform RTs and MTs in top-down MS, which contains an input layer, *k* (*k* = 1, 2, or 3) fully connected hidden layers with dropout for regularization, and a fully connected output layer. The 62 features in the GPTime model were the input for RT prediction, and 5 features were used for MT prediction: the 2 features in the semi-empirical model and the numbers of D, E, and N residues (see Results). For MT prediction, we normalized proteoform masses by dividing them by 20,000 and normalized proteoform charges by dividing them by 20. The rectified linear unit activation function was used for the hidden layers and the sigmoid function for the output layer. The model weights were initialized with a uniform distribution with zero mean and unit variance. The batch size was eight, the maximum training epochs was 12,000, the loss function was the mean squared error (MSE), and the optimizer was the Adam algorithm with a learning rate of 10^–6^. The early stopping strategy was applied during the training process with a patience of 100. Various dropout rates (0, 0.1, and 0.2) and node numbers (64, 128, 256, 512, and 1024) for the hidden layers were tested (Table S4 in the Supporting Information).

Three published neural network models were also assessed for predicting proteoform RTs and MTs in top-down MS: the CNN + capsule model in DeepRT+,^30^ the GRU + FNN model in Prosit,^32^ and the CNN + LSTM + FNN model in DeepDIA.^34^ In the three models, the loss function was the MSE, and the optimizer was Adam.^49^ The input of the CNN + capsule and CNN + LSTM + FNN models was the one-hot encoding of the amino acid sequence, and the input of the GRU + FNN model was a sequence of 20 integers representing the amino acid sequence. Zero padding was added to the right end of the sequence to obtain the same length of 200, which was longer than the maximum proteoform length in the data sets. The learning rates for the three models were the default value 0.001.

In the CNN + capsule model, the first two layers are convolutional ones, which are followed by two capsule layers connected by “dynamic routing” (Figure S1 in the Supporting Information). The root sum square of the output vector of the last capsule layer is reported as the predicted proteoform RT or MT. Various hyperparameter settings were evaluated for the batch size, the number of epochs, and the filter numbers and kernel sizes of the convolutional layers (Table S5 in the Supporting Information).

The GRU + FNN model consists of an embedding layer, a bidirectional GRU layer, a one-directional GRU layer, an attention layer, and two fully connected layers (Figure S2 in the Supporting Information). Hyperparameter settings, such as the unit number (64, 128, 256, and 512) in the GRU layers and the node number (64, 128, 256, and 512) in the dense layers, were tested to achieve the best prediction accuracy (Table S6 in the Supporting Information).

The CNN + LSTM + FNN model contains a convolutional layer, a max pooling layer, a bidirectional LSTM layer, and three dense layers (Figure S3 in the Supporting Information). A dropout layer with a rate of 0.5 is added between the LSTM and the first dense layer. We tuned the following hyperparameters of the model: the filter number and kernel size of the convolution layers, the number of units in the LSTM layer, and the number of features in the dense layers (Table S7 in the Supporting Information).

### 2.4 Calibration of RTs

Proteoform RT shifts between RPLC–MS runs in the ovarian tumor data were calibrated using the RT alignment with three steps. (1) Proteoform identifications in different RPLC–MS runs were matched using an RT alignment method in TopDiff (version 1.4.0), a tool in TopPIC suite.^44^ (2) The list of proteoforms identified and matched in all LC–MS runs were reported. Finally, (3) proteoform RTs of the 2nd to 10th runs were calibrated to match those in the first run using the proteoform list. To calibrate the RT of a proteoform *P* in the second run, we find the two neighboring proteoforms in the proteoform list, whose RTs are the closest to *P*: one neighboring proteoform is eluted before *P* and the other after *P*. The RTs of the neighboring proteoforms are mapped to those in the first run, and the calibrated RT of *P* is obtained by interpolation.

### 2.5 Calibration of MTs

Proteoform MT variations in the CZE-MS runs in the colon cancer cell data were removed by MT calibration^50^ with three steps. (1) Proteoform MTs were converted to their corresponding electrophoretic mobilities. (2) Variations in electrophoretic mobility were removed using the semi-empirical model in Section 2.3.2 and linear regression. Finally, (3) the resulting electrophoretic mobilities were converted back to calibrated MTs. Formula 1 in Section 2.3.2 was used for the conversion in the first and third steps. In the second step, proteoform electrophoretic mobilities in a CZE-MS run were predicted using the semi-empirical model. Then, a linear regression model *y* = *ax* + *b* was used to fit experimental mobilities *x* to mobilities *y* reported by the semi-empirical model in each run, where *a* and *b* are model parameters. For two CZE-MS runs, the electrophoretic mobilities of proteoforms in the second run were mapped to those in the first run using the following method. Let *a*_1_ and *b*_1_ be the regression parameters for the first run, and *a*_2_ and *b*_2_ be the regression parameters for the second run. For a proteoform with mobility *x* in the second run, its mobility *x̅* with calibration satisfies the equation *a*_1_*x̅* + *b*_1_ = *a*_2_*x* + *b*_2_, so the mobility with calibration is computed as (*a*_2_*x* + *b*_2_ – *b*_1_)/*a*_1_. To calibrate proteoform MTs in many runs, we choose one CZE-MS run as the reference and map proteoform MTs in other runs to those in the reference run.

### 2.6 Evaluation Criteria

Three metrics were selected to evaluate the performance of the machine learning models: the mean absolute error (MAE), Pearson correlation coefficient *R*, and Δ*t*_r95%_. The MAE measures the average error between predicted and empirical times, *R* measures the correlation between predicted and empirical times, and Δ*t*_r95%_ is the ratio between Δ*t*_95%_ and the overall elution/MT, where Δ*t*_95%_ is the minimal time window that explains 95% of the deviation between predicted and empirical times.

## 3 Results

### 3.1 Training and Test Data Sets

TopPIC identified 610 proteoforms of 188 proteins from the first replicate (LC-ONE) of the ovarian tumor RPLC–MS data. The proteoforms in the LC-ONE data were divided into 188 protein groups, which were then randomly split into a training set (131 protein groups with 437 proteoforms) and a test set (57 protein groups with 173 proteoforms) with a proteoform ratio of 7:3 approximately. We further combined PrSMs identified from the 10 replicates (LC-TEN) of the ovarian tumor RPLC-MS data and removed duplicated proteoforms (see Section 2.2), resulting in 1010 proteoforms of 265 proteins. The proteoform RTs were calibrated to map to those in the first run using RT alignment. The proteoforms in the LC-TEN were divided into 255 protein groups and randomly split into a training set (185 protein groups with 736 proteoforms) and a test set (80 protein groups with 274 proteoforms) with an approximate ratio of 7:3.

Similarly, TopPIC reported from the first replicate (CZE-ONE) of the CZE-MS/MS SW480 data 1230 proteoforms of 470 proteins, which were further randomly split by the protein group into a training set of 878 proteoforms from 329 proteins and a test set of 352 proteoforms from 141 proteins. We also combined proteoforms identified from all 36 CZE-MS runs (CZE-ALL) in the colon cancer cell data and removed duplicated ones, reporting 2914 proteoforms from 889 proteins. Then, we randomly split them into a training set (2105 proteoforms from 622 proteins) and a test set (809 proteoforms from 267 proteins) with an approximate ratio of 7:3.

The length distributions of the LC-ONE, LC-TEN, CZE-ONE, and CZE-ALL data sets are given in Figure S4 in the Supporting Information. The average proteoform lengths are 49, 53, 43, and 42 for the LC-ONE, LC-TEN, CZE-ONE, and CZE-ALL data sets, respectively.

### 3.2 RT and MT Calibration

We evaluated the effect of calibration on proteoform RT prediction accuracy using the LC-TEN data set and the GPTime model. Proteoform RTs in the 10 RPLC-MS runs were calibrated using the RT alignment and interpolation (see Methods). The GPTime model was trained and tested on the LC-TEN data set with the 7:3 training-test split using proteoform RTs before and after calibration. The prediction accuracy in the test data was similar for the RTs before calibration (*R* = 0.937 and MAE = 0.0470) and after calibration (*R* = 0.938, MAE = 0.0457), suggesting that there are only small RT shifts in the 10 replicate runs.

**Figure 1:**
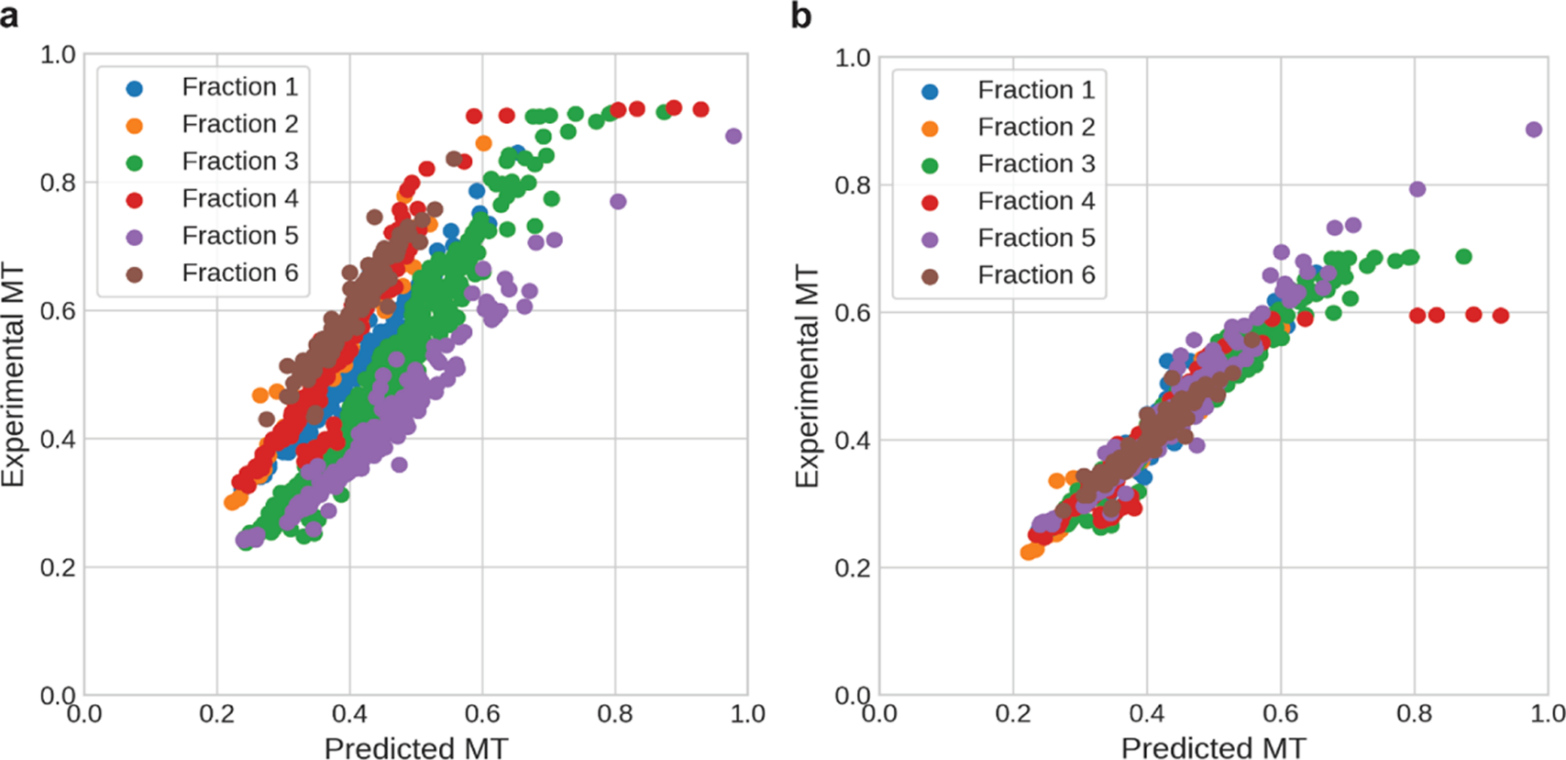
MT calibration for the CZE-ONE data set with prefractionation. (a) MTs predicted by the semi-empirical model are plotted against experimental MTs in six CZE-MS runs. The Pearson correlation coefficient between predicted and experimental MTs is 0.956 on average for single runs and 0.792 for the combined data of the six runs. (b) The Pearson correlation coefficient between predicted and experimental MTs is improved to 0.954 for the combined data after calibration.

The CZE-ONE data set contained proteoforms identified from six SEC fractions of the sample, and the measured proteoform MTs were affected by variations in the CZE-MS runs (Figure 1a). Because proteoform identifications in the fractions are different, time alignment^51^ is not a good choice for MT calibration of the data set. The semi-empirical model in Section 2.3.2 was applied to predict MTs for all proteoforms in the CZE-ONE data and performed well for single runs (average *R* = 0.956), but the variations in the runs for the six fractions affected its prediction accuracy (*R* = 0.792) for the combined data without calibration (Figure 1a). After calibration (see Methods), the Pearson correlation coefficient between experimental and predicted MTs was improved from 0.792 to 0.954 (Figure 1b), suggesting that calibration is an indispensable step for achieving high accuracy in proteoform MT prediction.

### 3.3 RT Prediction

To optimize the input features of LR, SVR, and RFR, the 62 features in GPTime were ranked based on the importance reported by a random forest regression model (number of trees: 350) trained on the LC-ONE training set (437 proteoforms of 131 protein groups). Using the top 10 features, the hyperparameters (not including the feature number) of SVR and RFR were tuned using fivefold cross-validation on the LC-ONE training set. The 131 protein groups were divided into five folds so that each fold contained approximately the same number of proteoforms. The best hyperparameter settings are given in Table S3. We then evaluated the accuracy of the LR, SVR, and RFR models with top *k* features (*k* = 1, 2, ..., 62) using the best hyperparameter settings and found that the best feature numbers for LR, SVR, and RFR were 28, 7, and 23, respectively. Hyperparameters were also tuned for the FNN, CNN + capsule, GRU + FNN, and CNN + LSTM + FNN models using the LC-ONE training set with fivefold cross-validation. The best hyperparameter settings for the four models are given in Tables S4–S7 in the Supporting Information.

Table 1 summarizes the prediction accuracy of LR, SVR, RFR, GPTime, and the four neural network models with the best hyperparameter settings on the LC-ONE and LC-TEN data sets with the 7:3 training-test split. The Pearson correlation coefficients of most of the models are between 0.92 and 0.94, and the prediction accuracies of traditional and neural network models are similar. The neural network models failed to achieve high accuracy as demonstrated in previous studies^30,32,34^ for peptide RT prediction owing to the small sizes of the training data sets. With the increase of the training data size from 437 (LC-ONE) to 736 (LC-TEN), the prediction accuracy of the CNN + capsule model slightly increases, while the accuracy of other neural network models is not significantly changed, indicating that the training data set of LC-TEN is still small for most neural network models to obtain high prediction accuracy.

**Table 1:** Benchmarking of Eight Machine Learning Models for Proteoform RT Prediction on the LC-ONE and LC-TEN Data Sets with the 7:3 Training-Test Split

Model	LC-ONE			LC-TEN		
	*R*	Δ*t*_r95%_	MAE	*R*	Δ*t*_r95%_	MAE
LR	0.922	0.468	0.0576	0.923	0.377	0.0576
SVR	0.911	0.518	0.0639	0.918	0.366	0.0587
RFR	0.935	0.423	0.0531	0.920	0.379	0.0565
GPTime	0.926	0.433	0.0535	0.938	0.337	0.0479
FNN	0.931	0.439	0.0534	0.913	0.378	0.0595
CNN + capsule	0.889	0.518	0.0699	0.920	0.395	0.0540
GRU + FNN	0.934	0.438	0.0516	0.929	0.385	0.0508
CNN + LSTM + FNN	0.913	0.443	0.0573	0.917	0.426	0.0534

### 3.4 MT Prediction

A total of seven proteoform features were divided into three groups and tested for proteoform MT prediction: the molecular mass and the charge state (group 1); the numbers of D, E, and N residues (group 2); and the numbers of L and I residues (group 3). A previous study^41^ of the semi-empirical model showed that the two features in group 1 are important for MT prediction and that D, E, and N residues (features in group 2) slightly influence the proteoform charge. The numbers of L and I residues (group 3 features) were selected owing to their high hydrophobicity indexes in CZE experiments.^52^ We compared four feature sets, which were used as the input of the FNN model with two hidden layers (256 nodes in each layer), on the CZE-ONE training set with fivefold cross-validation: (1) group 1 only, (2) groups 1 and 2, (3) groups 1 and 3, and (4) all the features. The FNN model with the features in groups 1 and 2 obtained the best prediction accuracy *R* = 0.981 (Table S8 in the Supporting Information), suggesting that the features in group 2 provided additional information for MT prediction.

Hyperparameter settings of the four neural network models were tuned using the CZE-ONE training set with fivefold cross-validation. The best hyperparameter settings of the models are given in Tables S4–S7 in the Supporting Information. The best hyperparameter settings for the models are not the same as those for RT and MT prediction, which is reasonable because the RPLC and CZE separation methods are different. We tested the prediction accuracy of the semi-empirical model and four neural network models with two experimental settings: the 7:3 training-test split of the CZE-ONE data set and the 7:3 training-test split of the CZE-ALL data set. Experimental results showed that the performance of the GRU + FNN and FNN models slightly outperformed other models on the two data sets (Table 2). The semi-empirical and FNN models reported high prediction accuracy with several proteoform features, indicating that it is possible to accurately predict proteoform MTs with simple models. Increasing the training data size from 878 (CZE-ONE) to 2105 (CZE-ALL) significantly improved the prediction accuracy of CNN + capsule and CNN + LSTM + FNN, showing that complex models need a large training data set to obtain high prediction accuracy.

**Table 2:** Benchmarking of the Semi-Empirical Model and Four Neural Network Models for Proteoform MT Prediction on the CZE-ONE and CZE-ALL Data Sets with the 7:3 Training-Test Split

	CZE-ONE			CZE-ALL		
Model	*R*	Δ*t*_r95%_	MAE	*R*	Δ*t*_r95%_	MAE
semi-empirical	0.953	0.185	0.0179	0.970	0.141	0.0130
FNN	**0.975**	**0.130**	**0.0137**	**0.981**	**0.113**	**0.0107**
CNN + capsule	0.865	0.293	0.0329	0.946	0.207	0.0206
GRU + FNN	**0.973**	**0.127**	**0.0119**	**0.982**	**0.102**	**0.0106**
CNN + LSTM + FNN	0.777	0.387	0.0445	0.969	0.145	0.0142

### 3.5 Transfer Learning

**Figure 2:**
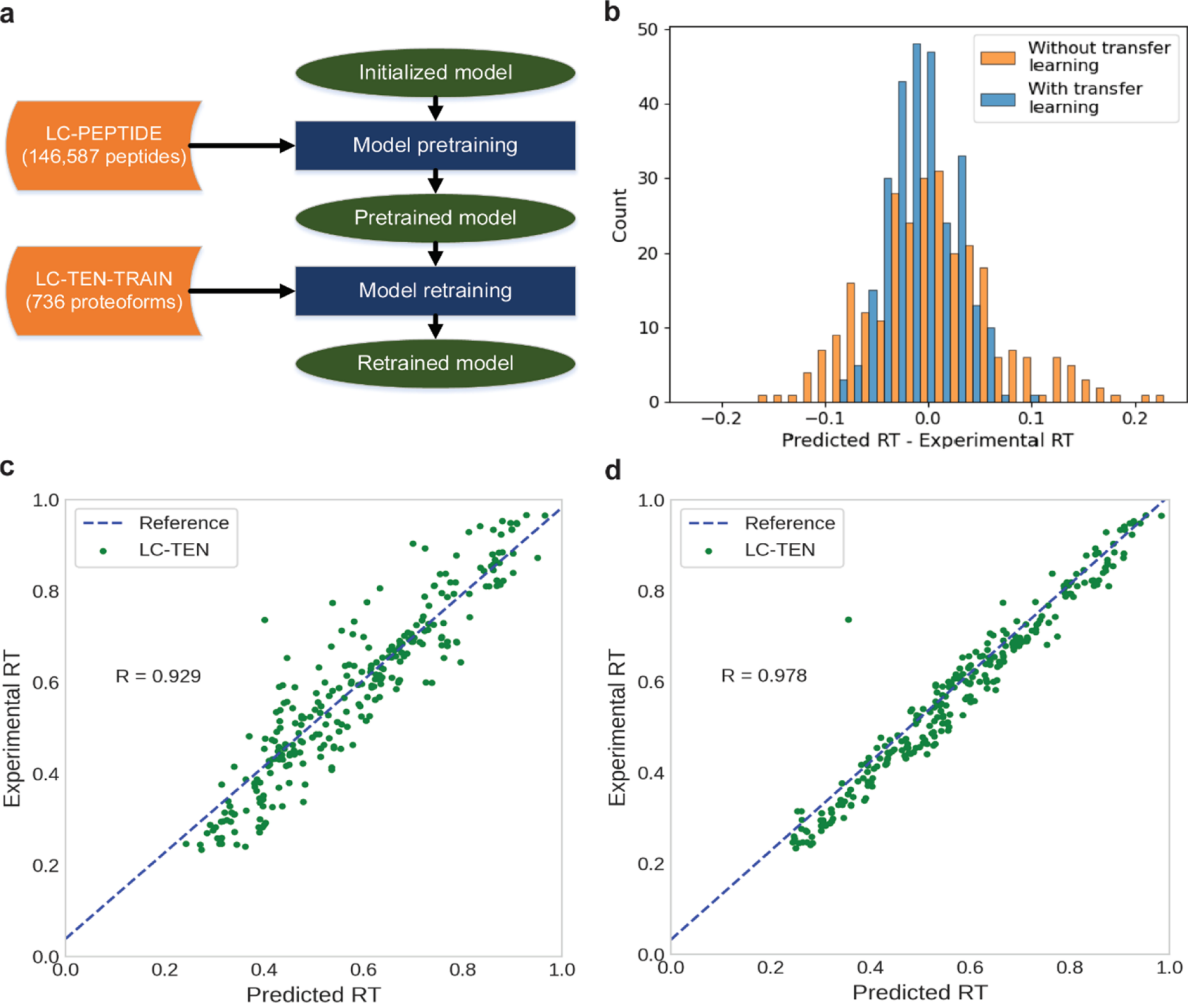
Comparison of the GRU + FNN model with and without transfer learning on the LC-TEN data. (a) An overview of the transfer learning method with the LC-PEPTIDE data for pretraining and the LC-TEN training data set for retraining. (b) Histograms of proteoform RT prediction errors for the model trained with and without transfer learning on the LC-TEN test data. (c) The Pearson correlation coefficient of the GRU + FNN model is 0.929 when it is trained with the LC-TEN training set and tested on the LC-TEN test set. (d) The Pearson correlation coefficient of the GRU + FNN model is 0.978 when it is pretrained using the LC-PEPTIDE data, retrained with the LC-TEN training set, and tested on the LC-TEN test set.

Transfer learning^53^ was adopted to address the problem that a large training data set was lacking for proteoform RT and MT prediction. The main idea of transfer learning is to combine peptide data in bottom-up MS and proteoform data in top-down MS to train machine learning models. The four neural network models were first pretrained with a large data set of peptides with RTs or MTs identified by bottom-up MS, and then, the learned knowledge was transferred to the retraining of the models with proteoform data by initializing the model parameters with the values obtained from pretraining (Figure 2a). The hyperparameters of the models were the same as those in Tables S4 and S7. The models for RT prediction were pretrained using a bottom-up RPLC–MS/MS data set of 24 human cell lines and tissues.^54^ X!Tandem^55^ identified 146,587 unique peptides, referred to as LC-PEPTIDE, from the data set using database search, and the iRT Toolkit^54^ reported normalized RTs of the identified peptides. Detailed methods for identifying peptides and obtaining RTs can be found in the study by Escher et al.^54^ The four neural network models were assessed on the LC-TEN test data set using three training methods: (1) pretraining with the LC-PEPTIDE data, (2) training with the LC-TEN training data only, and (3) pretraining with the LC-PEPTIDE data and retraining with the LC-TEN training data. In addition, linear regression was employed to fit the RTs predicted by the first training method to experimental RTs. The transfer learning method increased the prediction accuracy of all the four neural network models compared with the other two training methods (Table 3, Figures S5a and S7). Specifically, the GRU-FNN model achieved a prediction accuracy of *R* = 0.974 with only peptide pretraining; the transfer learning method reduced the prediction errors of many proteoforms (Figure 2b) and improved its prediction accuracy from *R* = 0.929 to 0.978 (Figure 2c,d) compared with the second training method, indicating that the knowledge obtained from peptide data can be efficiently transferred to the retraining step for the model. The prediction accuracy of the FNN model was not significantly improved by the transfer learning method, which might be due to its simple architecture.

**Table 3:** FNN, CNN + Capsule, GRU + FNN, and CNN + LSTM + FNN Models Are Assessed on the LC-TEN Test Data Using Three Training Methods: (1) Pretraining Using the LC-PEPTIDE Data Only, (2) Training Using the LC-TEN Training Data Only, and (3) Transfer Learning: Pretraining Using the LC-PEPTIDE Data and Retraining with the LC-TEN Training Data

Model	pretraining with LC-PEPTIDE data			training with LC-TEN training data			transfer learning		
	*R*	Δ*t*_r95%_	MAE	*R*	Δ*t*_r95%_	MAE	*R*	Δ*t*_r95%_	MAE
FNN	0.914	0.385	0.0573	0.913	0.378	0.0595	**0.933**	**0.352**	**0.0518**
CNN + capsule	0.767	0.756	0.0820	0.920	0.395	0.0540	**0.951**	**0.279**	**0.0415**
GRU + FNN	0.974	0.180	0.0279	0.929	0.385	0.0508	**0.978**	**0.172**	**0.0271**
CNN + LSTM + FNN	0.845	0.607	0.0576	0.917	0.426	0.0534	**0.965**	**0.240**	**0.0326**

The four neural network models for MT prediction were pretrained using a bottom-up CZE-MS/MS data set of HeLa cells.^56^ The data set was generated from tryptic digestion of proteins of HeLa cells, and the spectra in the data set were analyzed by Mascot^57^ (version 2.2.4) in Proteome Discoverer 1.4 for peptide identification. We filtered out all identified peptides with PTMs or with a *q*-value >0.001, resulting in 4234 unique peptide identifications, referred to as CZE-PEPTIDE. The MTs of the peptides were obtained from the LC–MS data using Mascot. Similar to proteoform RT prediction, we evaluated the four neural network models on the CZE-ALL test data using three training methods: (1) pretraining with the CZE-PEPTIDE data only, (2) training with the CZE-ALL training data only, and (3) pretraining with the CZE-PEPTIDE data and retraining with the CZE-ALL training data. The transfer learning method slightly improved the prediction accuracy for the CNN + capsule model but failed to significantly increase the accuracy for the other three models (Table 4, Figures S5b and S8). The reason might be that the CZE-ALL training data were enough to achieve a high prediction accuracy for the models and that pretraining could provide only limited additional information.

**Table 4:** FNN, CNN + Capsule, GRU + FNN, and CNN + LSTM + FNN Models Are Evaluated on the CZE-ALL Test Data Using Three Training Methods: (1) Pretraining Using the CZE-PEPTIDE Data Only, (2) Training Using the CZE-ALL Training Data Only, and (3) Transfer Learning: Pretraining Using the CZE-PEPTIDE Data and Retraining with the CZE-ALL Training Data

Model	pretraining with CZE-PEPTIDE data			training with CZE-TEN training data			transfer learning		
	*R*	Δ*t*_r95%_	MAE	*R*	Δ*t*_r95%_	MAE	*R*	Δ*t*_r95%_	MAE
FNN	0.965	0.152	0.0169	**0.981**	**0.113**	**0.0107**	0.980	0.109	0.0109
CNN + capsule	0.865	0.302	0.0314	0.946	0.207	0.0206	**0.971**	**0.142**	**0.0146**
GRU + FNN	0.943	0.210	0.0237	**0.982**	**0.102**	**0.0106**	0.982	0.103	0.0104
CNN + LSTM + FNN	0.343	0.595	0.0651	0.969	0.145	0.0142	**0.977**	**0.123**	**0.0124**

We further investigated if a small peptide pretraining data set can improve proteoform RT prediction using transfer learning. We randomly selected 4234 out of the 146,587 peptides in the LC-PEPTIDE data set for pretraining and tested the GRU + FNN model on the LC-TEN data set with transfer learning. The trained model obtained a prediction accuracy of *R* = 0.971 and MAE = 0.0305 on the LC-TEN test data, which is similar to the performance with all the peptides for pretraining (*R* = 0.978 and MAE = 0.0271) (Table S9).

### 3.6 RT Prediction for Long Proteoforms

**Figure 3:**
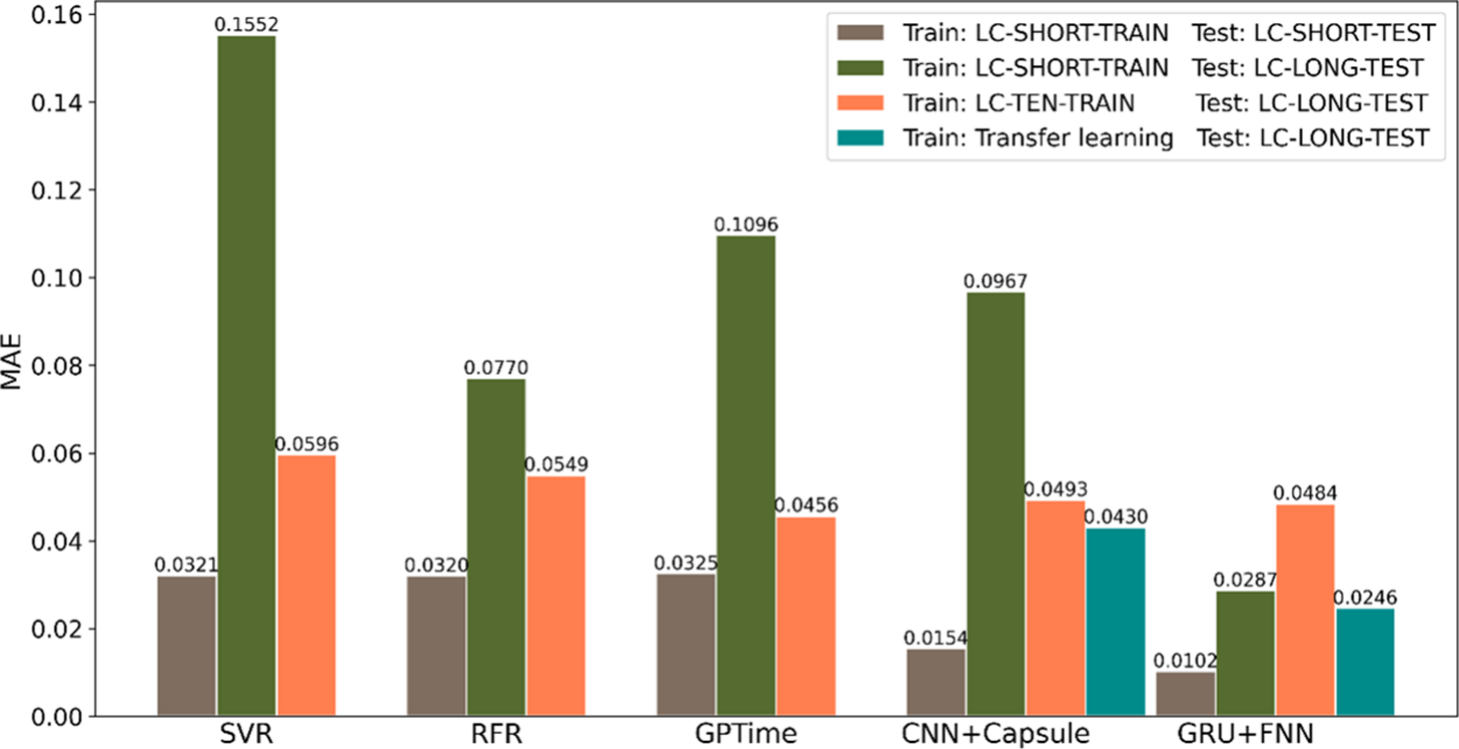
Comparison of the MAEs of SVR, RFR, GPTime, CNN + capsule, and GRU + FNN using four training and test methods. (1) Training with the LC-SHORT training data and testing on the LC-SHORT test data; (2) training with the LC-SHORT training data and testing on the LC-LONG-TEST data; (3) training with the LC-TEN training data and testing on the LC-LONG-TEST data; and (4) transfer learning with the LC-SHORT training data for pretraining and the LC-TEN training data for retraining and testing on the LC-LONG-TEST data. The fourth method is used for CNN + capsule and GRU + FNN only.

We assessed the RT prediction accuracy of SVR, RFR, GPTime, CNN + capsule, and GRU + FNN for peptides with length <40 and proteoforms with length ≥40. The LC-PEPTIDE data used in transfer learning contain 145,714 peptides with <40 amino acids, referred to as LC-SHORT, which was randomly split into a training set with 101,999 peptides and a test set with 43,715 peptides with a ratio of 7:3. The LC-TEN test set contains 146 proteoforms with ≥40 amino acids, referred to as LC-LONG-TEST. We first trained the five models using the LC-SHORT training set and tested them on the LC-SHORT test set and the LC-LONG-TEST data. The CNN + capsule and GRU + FNN models trained with the LC-SHORT training data achieved high prediction accuracies (CNN + capsule: *R* = 0.995, GRU + FNN: *R* = 0.996) on the LC-SHORT test data, which is similar to the results reported previously.^30,32^Figure 3 shows that the MAEs of the models on the LC-LONG-TEST data are much higher than those on the LC-SHORT test set, revealing that the models trained using peptides tend to have large errors for RT prediction of long proteoforms. We also trained the five models using the LC-TEN training data and trained the CNN + capsule and GRU + FNN models using transfer learning: pretraining the models using LC-SHORT training data and retraining using the LC-TEN training data. The trained models were tested on the LC-LONG-TEST data and achieved much lower MAEs compared with the models trained with the LC-SHORT training data, suggesting that training or retraining with long proteoforms could help learn characteristics specific to long proteoforms for RT prediction.

### 3.7 Proteoform Identification with RT/MT Prediction

**Figure 4:**
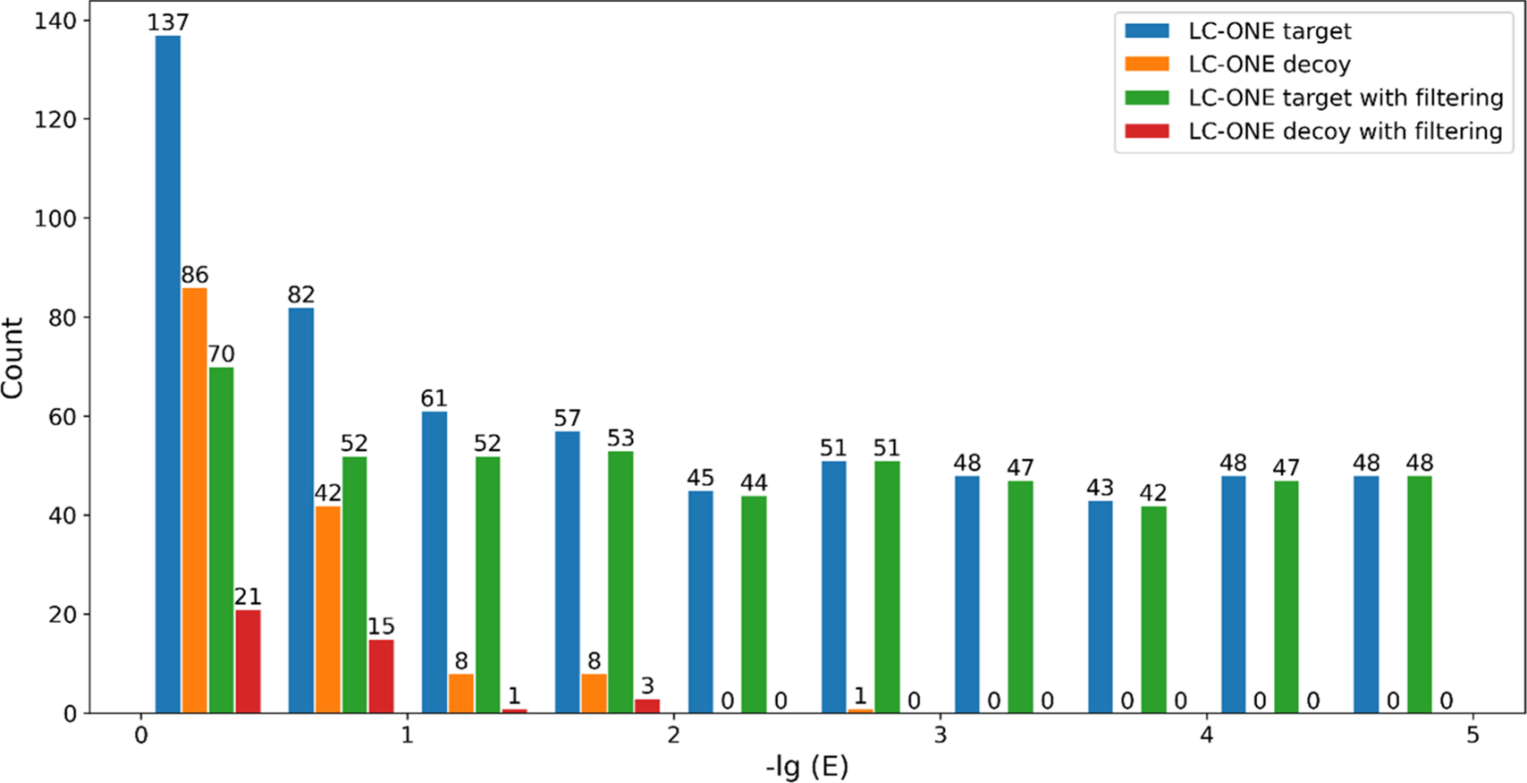
Filtering proteoform identifications using the differences between experimental and theoretical RTs reported by the GRU + FNN model. Target and decoy proteoforms identified from the LC-ONE data with an *E*-value <1 are filtered with a cutoff value of 0.1 for experimental and theoretical RT differences. The numbers of target and decoy proteoforms are plotted against their *E*-values with logarithm transformation.

We evaluated if RT/MT prediction with the GRU + FNN model can increase the number of proteoform identifications. An incorrect proteoform identification in top-down MS tends to have a large difference between its experimental and theoretical RTs or MTs (Figure S6). Therefore, the quality of a proteoform identification is evaluated by its *E*-value reported by TopPIC and the difference between its experimental and theoretical RTs/MTs predicted by the GRU + FNN model. Figure 4 illustrates that RT differences are important for filtering out decoy identifications with an *E*-value ≥0.01. Based on the observation, we filtered out proteoforms with an *E*-value ≥0.01 and a theoretical and experimental RT difference ≥0.1 identified from the LC-ONE data set. After the filtering method was added to TopPIC, with a 1% proteoform-level FDR, the number of proteoform identifications of the LC-ONE data set was increased from 1090 to 1154 (5.9%), and the number of protein identifications was increased from 291 to 305 (4.8%). The filtering method also increased the number of proteoform identifications from 2146 to 2166 (1.0%) and the number of protein identifications from 741 to 749 (1.1%) with a 1% proteoform-level FDR for the CZE-ONE data set. The RTs and MTs predicted by the GRU + FNN model (Figures 4 and S9) are not accurate enough to separate target identifications from decoy ones to achieve an FDR of 1%, which is the reason that the increase in the number of proteoform identifications was limited. For decoy identifications, the differences between theoretical and experimental MTs are smaller than those between theoretical and experimental RTs (Figure S6), so the increase of proteoform identifications for the CZE-ONE data was less significant than that for the LC-ONE data. The proteoform charge and molecular mass are two dominant features in the MT prediction models. The molecular masses of identified decoy proteoforms are not randomly distributed. If a decoy proteoform is matched to a query spectrum, then its molecular mass is similar to that of the proteoform from which the query spectrum was generated. Because of this, the MT prediction errors of identified decoy proteoforms follow a Gaussian-like distribution. Additionally, the distributions of the charges of identified target and decoy proteoforms are different: identified decoy proteoforms tend to have higher charges than identified target proteoforms (Figure S10). As a result, the distribution of the MT prediction errors of identified decoy proteoforms is skewed to the left compared with identified target proteoforms (Figure S6b).

## 4 Discussion

The GRU + FNN model designed for peptide RT prediction in bottom-up MS achieved an accuracy of *R* = 0.978 for proteoform RT prediction and *R* = 0.982 for proteoform MT prediction with transfer learning, demonstrating that it is not significantly affected by long proteoforms with ≥40 amino acids (Figures S4 and S5). The GRU^58^ and attention layers^59^ in the GRU + FNN model are designed for processing long sequences, so it might be inheritably suitable for proteoform RT and MT prediction. The simple two-gate structure in GRU might be the reason that the GRU + FNN model could be efficiently trained with a small data set without transfer learning. The prediction accuracy of the CNN + capsule and CNN + LSTM + FNN models without transfer learning dropped significantly for RT prediction in top-down MS compared with that in bottom-up MS owing to limited training data. The prediction accuracies of these two models were improved when bottom-up data were used for pretraining in transfer learning, suggesting that large training data are essential to improving their prediction accuracy.

The four neural network models reported comparable prediction accuracy for proteoform RT prediction in RPLC and MT prediction in CZE, showing that these models have strong generality for prediction problems in proteoform separation and may be used for other prediction problems, such as SEC and IEC RT prediction. With only several features, including proteoform mass and charge, the semi-empirical and FNN models obtained a high accuracy for proteoform MT prediction, and most of the models reported a higher accuracy for MT prediction than RT prediction, suggesting that RT prediction is more complicated than MT prediction.

Because of the similarity between peptides and proteoforms, the GRU + FNN model trained on peptide data can be used to predict proteoform RTs and MTs with calibration. Transfer learning in general can further improve the prediction accuracy of a model for proteoform RT and MT prediction by first pretraining the model on a large data set obtained from bottom-up MS and then retraining the model using a top-down MS data set. However, it may fail to improve prediction accuracy in some cases, such as the GRU + FNN model for MT prediction (Table 4). The performance of transfer learning may depend on the model architecture, the sizes of the bottom-up and top-down data sets, and whether there exists information that is transferable and indispensable from the pretraining data.

The study of the CZE-ONE data with prefractionation reveals that the variations in CZE runs significantly affect experimental MTs and that calibration is an indispensable step for accurate prediction. Most of the variations in CZE runs can be removed by a regression-based method. The existence of variations also complicates the application of RT and MT prediction models: a model trained on one data set needs to be calibrated or retrained before it is used on another data set.

RT and MT prediction can increase proteoform identification in top-down MS. When proteoforms lack confident spectral identification, RT and MT prediction becomes more important for proteoform identification. However, when the accuracy is not high enough, the improvement for proteoform identification is limited.

There are still many challenges in proteoform RT and MT prediction. The first challenge is that there is a lack of large data sets for training complex machine learning models owing to the low proteoform coverage of top-down MS. One possible solution is to combine proteoforms identified from multiple species with the same MS experimental setting. The second challenge is to predict RTs and MTs of modified proteoforms. The number of identified proteoforms with a specific PTM is even lower than unmodified proteoforms. The third challenge is how to apply trained machine learning models to MS data sets generated with various settings, which can cause shifts in RTs or MTs of proteoforms.

## 5 Conclusions

In this paper, we assessed several machine learning models for proteoform RT and MT prediction in top-down MS. The GRU + FNN model in Prosit with transfer learning achieved high accuracy for proteoform RT prediction, and the GRU + FNN and FNN models outperformed other models in proteoform MT prediction. Experimental results on transfer learning also showed its potential to increase prediction accuracy by using peptides identified from bottom-up MS for pretraining. In future work, we will generate large training data sets, further improve RT and MT prediction accuracy, and study the RT and MT prediction problems for modified proteoforms.

## Supporting Information Available

Summary of deep learning models for peptide RT prediction; parameter settings of TopPIC; hyperparameter settings for LR, SVR, and RFR; hyperparameter settings for the FNN model; hyperparameter settings for the CNN + capsule model; hyperparameter settings for the GRU + FNN model; hyperparameter settings for the CNN + LSTM + FNN model; performance of the FNN model with four feature sets for MT prediction on the CZE-ONE data set with fivefold cross-validation; comparison of transfer learning for RT prediction with different sizes of pretraining data sets; architecture of the DeepRT+ (CNN + capsule) model; architecture of the Prosit (GRU + FNN) model; architecture of the DeepDIA (CNN + LSTM + FNN) model; histograms of the proteoform length in four data sets; comparison of the differences between predicted and experimental times for the GRU + FNN with only bottom-up peptide data pretraining and with transfer learning; comparison of the differences between predicted and experimental times of identified target and decoy proteoforms; comparison of RT prediction performance on the LC-TEN data set; comparison of MT prediction performance on the CZE-ALL data set; filtering proteoform identifications using the differences between experimental and theoretical MTs reported by the GRU + FNN model; and charge distributions of target and decoy proteoforms identified from the CZE-ONE data set (PDF)

## Notes

Availability: The code and data sets are available at https://github.com/wenronchen/rt_prediction.
