# Supplementary material for: Evaluation of Machine Learning Models for Proteoform Retention and Migration Time Prediction in Top-Down Mass Spectrometry
Source: J Proteome Res. 2022 May 26;21(7):1736–47. doi: 10.1021/acs.jproteome.2c00124 (PMC9250612; doi:10.1021/acs.jproteome.2c00124)
Supplement: Supplementary file 1 — pr2c00124_si_001.pdf [file pr2c00124_si_001.pdf]

Evaluation of machine learning models for proteoform retention and migration time prediction in top-down mass spectrometry (supplementary material)

Wenrong Chen[1], Elijah N. McCool[2], Liangliang Sun[2], Yong Zang[3], Xia Ning[4,5,6], and Xiaowen Liu[7,8]*

[1]Department of BioHealth Informatics, Indiana University-Purdue University Indianapolis, Indianapolis, IN 46202, USA, [2]Department of Chemistry, Michigan State University, East Lansing, MI 48824, USA, [3]Department of Biostatics and Health Data Sciences, Indiana University School of Medicine, Indianapolis, IN, 46202, USA, [4]Department of Biomedical Informatics, The Ohio State University, Columbus, Ohio 43210, USA, [5]Department of Computer Science and Engineering, The Ohio State University, Columbus, Ohio 43210, USA, [6]Translational Data Analytics Institute, The Ohio State University, Columbus, Ohio 43210, USA, [7]Tulane Center for Biomedical Informatics and Genomics, Tulane University, New Orleans, LA, 70112, USA, [8]Deming Department of Medicine, Tulane University, New Orleans, LA 70112, USA

*Corresponding author: 1441 Canal St, New Orleans, LA 70112, xwliu@tulane.edu

Table of Contents

**Tables**	**Description**
Table S1	A summary of deep learning models for peptide retention time prediction
Table S2	Parameter settings of TopPIC
Table S3-S7	Hyperparameter settings for models
Table S8	Performance of the FNN model with 4 feature sets for MT prediction on the CZE-ONE data set with 5-fold cross-validation
Table S9	Comparison of transfer learning for RT prediction with different sizes of pretraining data sets
**Figures**	
Figure S1	The architecture of the DeepRT+ (CNN+Capsule) model
Figure S2	The architecture of the Prosit (GRU+FNN) model
Figure S3	The architecture of the DeepDIA (CNN+LSTM+FNN) model
Figure S4	Histograms of the proteoform length in four data sets
Figure S5	Comparison of the differences between predicted and experimental times for the GRU+FNN with only bottom-up peptide data pretraining and with transfer learning
Figure S6	Comparison of the differences between predicted and experimental times of identified target and decoy proteoforms
Figure S7	Comparison of RT prediction performance on the LC-TEN data set
Figure S	Comparison of MT prediction performance on the CZE-ALL data set
Figure S9	Filtering proteoform identifications using the differences between experimental and theoretical MTs reported by the GRU+FNN model
Figure S10	The charge distributions of target and decoy proteoforms
**References**	

Tables

Table S1. A summary of deep learning models for peptide retention time prediction

Model	Data set	Type	No. of peptides	Accuracy*
DeepRT (+)[1]	Yeast	RPLC	14361	R^2=0.99
DeepLC[2]	HeLa	RPLC	161193	R=0.99
Prosit[3]	Human	HPLC	219089	R=0.99
DeepMass[4]	Human plasma, HeLa, yeast	LC	69680	R^2=0.97
DeepDIA[5]	HeLa	RPLC	69577	R=0.99
AutoRT[6]	HeLa	LC	136791	MAE=0.47

*R^2 represents the square of correlation coefficient between experimental and predicted retention times.

Table S2. Parameter settings of TopPIC

Parameter	Value
Number of combined spectra	1
Fragmentation method	FILE
Search type	TARGET+DECOY
Fixed modifications	None/C57
Use TopFD feature file	TRUE
Maximum number of unexpected modifications	0
Error tolerance	15 ppm
Spectrum-level cutoff type	FDR
Spectrum-level cutoff value	0.05
Proteoform-level cutoff type	FDR
Proteoform-level cutoff value	0.05
Allowed N-terminal forms	NONE, NME, NME_ACETYLATION, M_ACETYLATION
Maximum mass shift of modifications	500 Da
Minimum mass shift of modifications	-500 Da
Thread number	15
E-value computation	Generation function

Table S3. Hyperparameter settings for LR, SVR, and RFR

Model	Parameter settings
Linear Regression	Intercept fitting: yes
Support Vector Regression	Kernel type: linear; L2 regularization coefficient: 30
Random Forest Regression	Number of trees: 150

Table S4. Hyperparameter settings for the FNN model

Parameter	Search space	Setting for RT prediction	Setting for MT prediction
#Hidden layers	[1,2,3,4]	3	2
#Dense features	[64,128,256,512,1024]	128	256
Dropout rate	[0,0.1,0.2]	0	0

Table S5. Hyperparameter settings for the CNN+Capsule model

Parameter	Search space	Setting for RT prediction	Setting for MT prediction
CNN filter number	[64,128,256]	128	128
Kernel size	[4,6,8,10,12,14,16,18,20]	8	16
Batch normalization	True/False	False	False
Batch size	(8,20)	16	19
#Epochs	[20,30,40,50,60]	40	40

Table S6. Hyperparameter settings for the GRU+FNN model

Parameter	Search space	Setting for RT prediction	Setting for MT prediction
Embedding dimension	[20,24,28,32]	24	24
#GRU units	[64,128,256,512,1024]	512	128
#Dense features	[64,128,256,512]	256	256

Table S7. Hyperparameter settings for the CNN+LSTM+FNN model

Parameter	Search space	Setting for RT prediction	Setting for MT prediction
CNN Filter number	[8,16,32,64,128]	64	16
Kernel size	[2,3,4,5]	4	3
#LSTM features	[16,32,64,128,256]	64	64
#Dense features	[32,64,128,256,512]	128	64

Table S8. Performance of the FNN model with 4 feature sets for MT prediction on the CZE-ONE data set with 5-fold cross-validation. A total of 7 features are divided into 3 groups: (1) the molecular mass and the charge state, (2) the numbers of D, E, and N residues, and (3) the numbers of L and I residues.

Features	*R*	MAE
Group 1	0.978	0.0129
Groups 1 and 2	**0.981**	**0.0122**
Groups 1 and 3	0.978	0.0127
Groups 1, 2, and 3	0.980	0.0122

Table S9. Comparison of transfer learning for RT prediction with different sizes of pretraining data sets. The GRU+FNN model is pretrained with a peptide data set, retrained with the LC-TEN training data set, and tested on the LC-TEN test data set.

Pretraining data	*R*	MAE
146587 peptides	0.978	0.0271
4234 peptides	0.971	0.0305

Figures

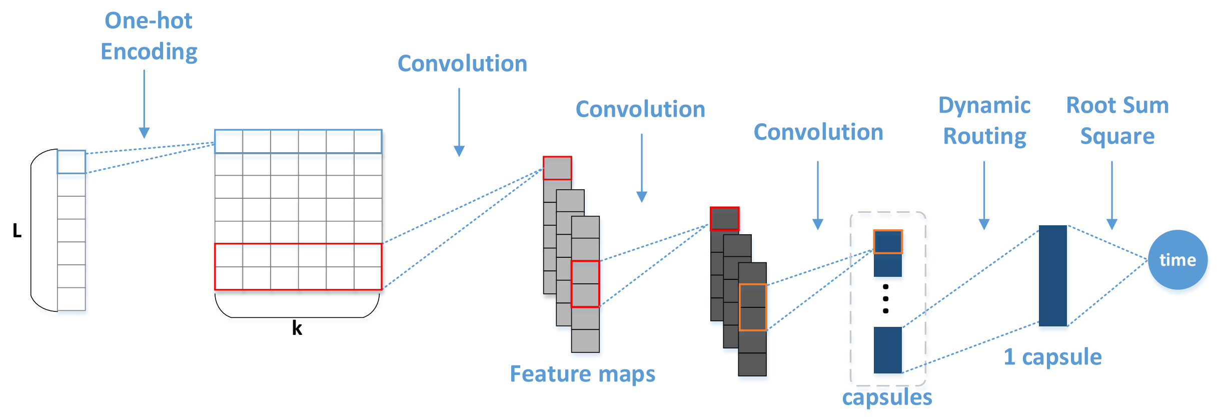

Figure S1. The architecture of the DeepRT+[1] (CNN+Capsule) model. The input protein sequence (length= L) is encoded with one-hot encoding to an $L \times k$ matrix where L is the padded length (200) of the sequence and k = 20 is the number of different types of amino acid residues. The encoded matrix is fed into two convolutional layers followed by two capsule layers with dynamic routing. The root sum square (RSS) of the output vector of the final capsule is reported.

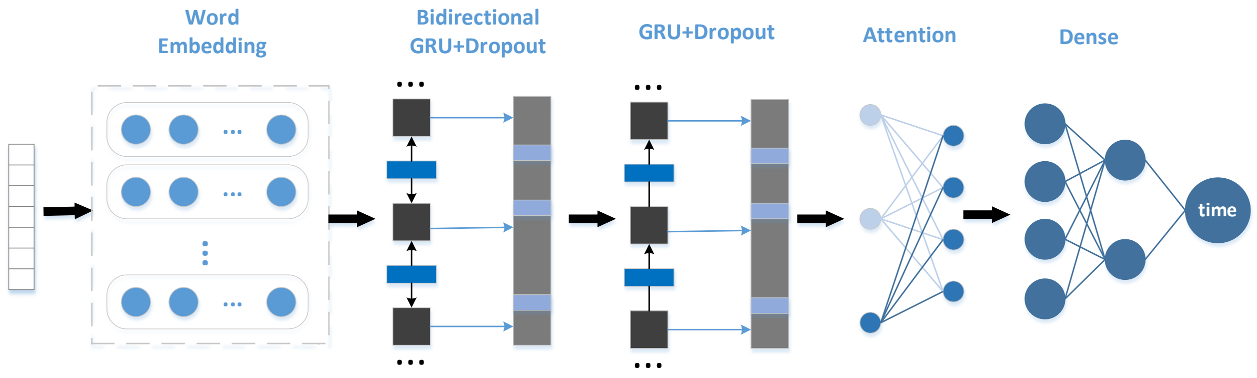

Figure S2. The architecture of the Prosit[3] (GRU+FNN) model. The input protein sequence is encoded by a word embedding layer, which is connected to one bidirectional recurrent layer with Gated Recurrent Unit (GRU) and one normal recurrent layer with GRU. The output from the dropout layer is flattened with an attention layer, which is connected to two dense layers.

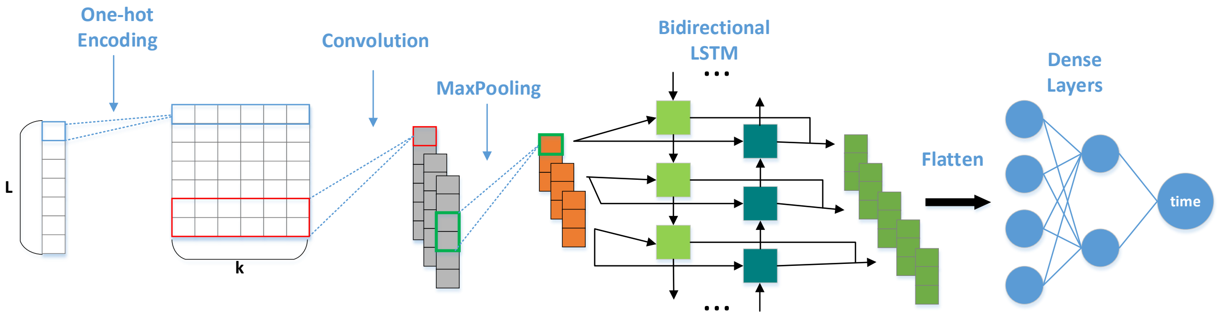

Figure S3. The architecture of the DeepDIA[5] (CNN+LSTM+FNN) model. The input sequence (length= *L*) is encoded with one-hot encoding to an *L*×*k* matrix, which *k* is number of different types of amino acids. The input features are fed into a convolutional layer with max pooling. A bidirectional LSTM layer is used to capture the sequential patterns in the output of max pooling. The output of the bidirectional LSTM layer is flattened, and two dense layers are used to generate the final prediction result.

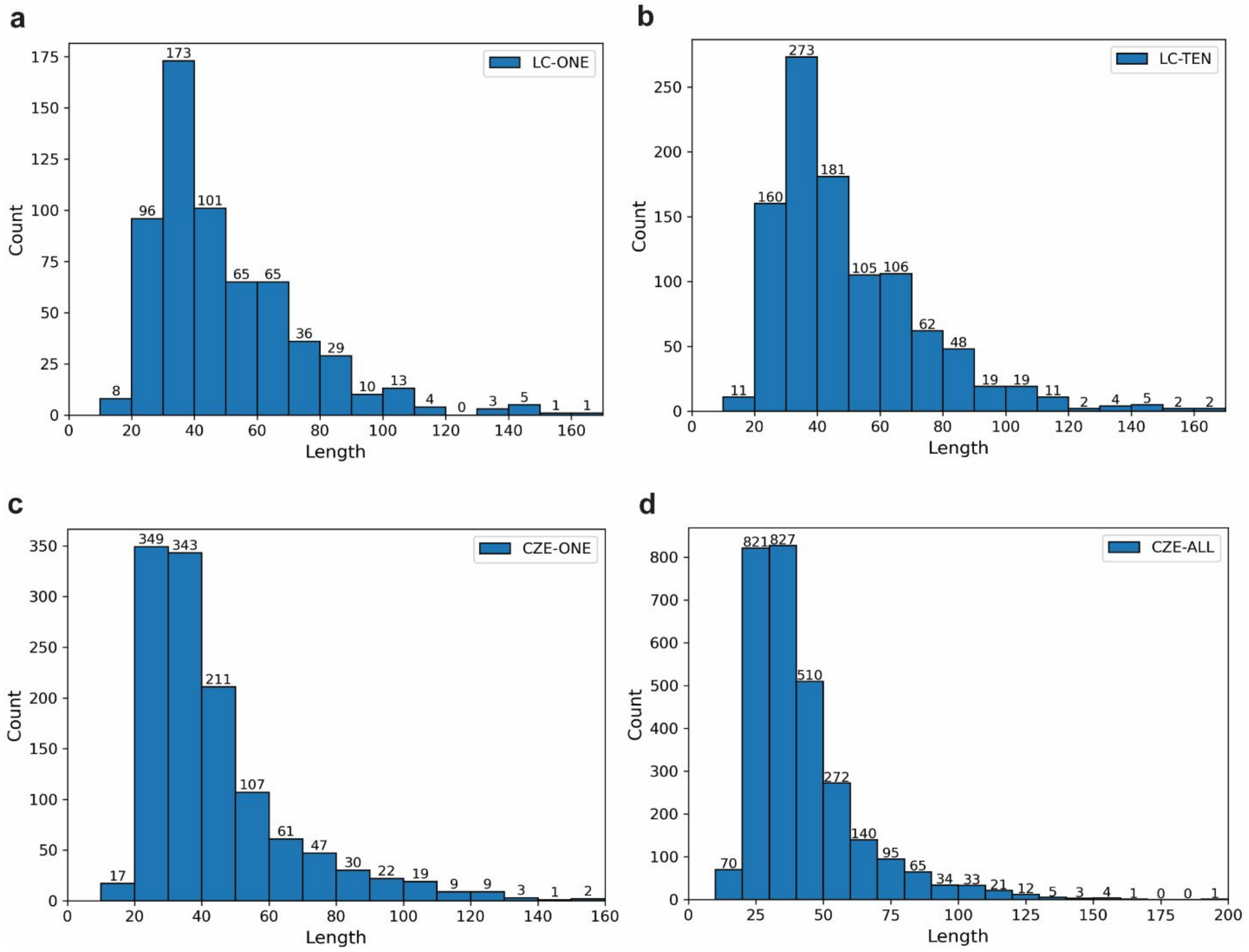

Figure S4. Histograms of the proteoform length in four data sets. (a) LC-ONE with an average length of 49. (b) LC-TEN with an average length of 53. (c) CZE-ONE with an average length of 43. (d) CZE-ALL with an average length of 42.

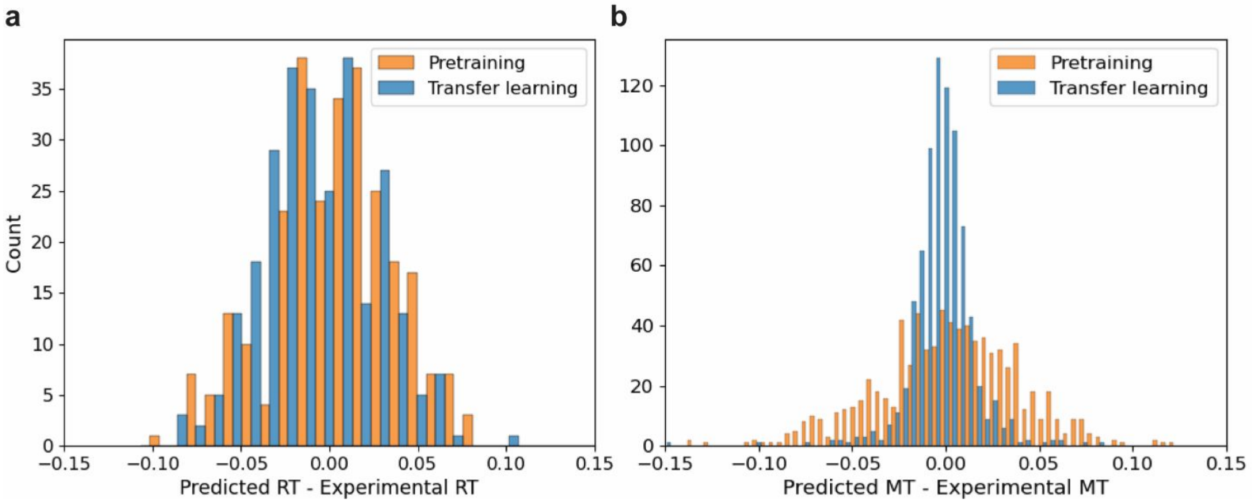

Figure S5. Comparison of the errors between predicted and experimental times for the GRU+FNN with only bottom-up peptide data pretraining and with transfer learning. (a) Histogram of the errors between predicted and experimental RTs for the LC-TEN test data set. The GRU+FNN model is pretrained with the LC-PEPTIDE data and retrained with the LC-TEN training data set. (b) Histogram of the errors between predicted and experimental MTs for the CZE-ALL test data. The GRU-FNN model is pretrained with the CZE-PEPTIDE data and retrained with the CZE-ALL training data. The retraining step reduces the MAE by 3.0% for RT prediction and 56.1% for MT prediction.

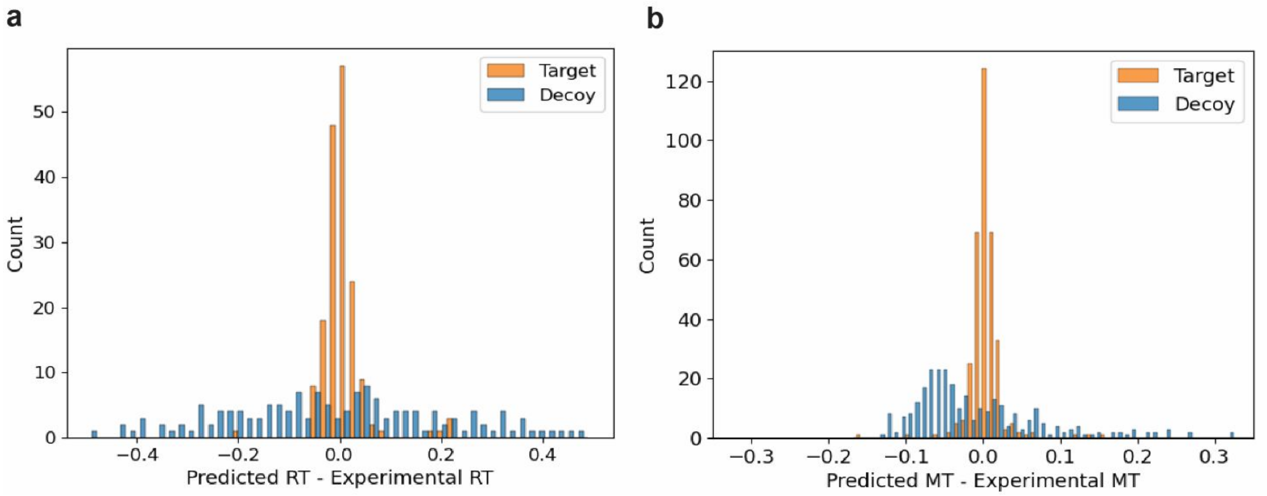

Figure S6. Comparison of the differences between predicted and experimental times of identified target and decoy proteoforms. (a) Histogram of the RT prediction differences for target and decoy proteoforms reported from the LC-ONE data set with an *E*-value cut-off of 1. (b) Histogram of the MT prediction differences for target and decoy proteoforms reported from the CZE-ONE data set with an *E*-value cut-off of 1.

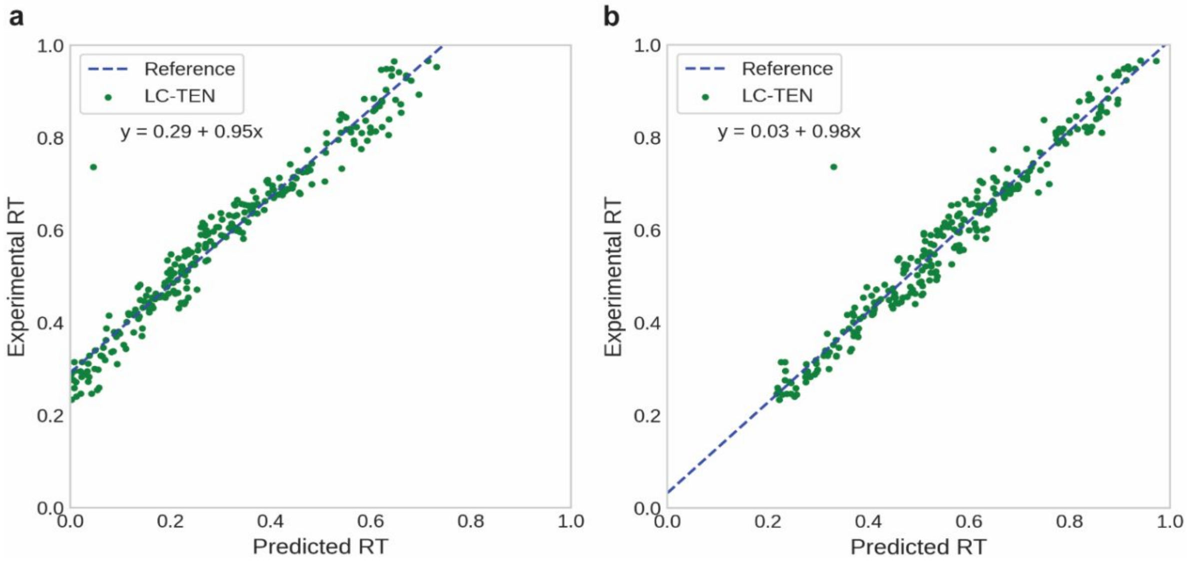

Figure S7. Comparison of RT prediction accuracy of the GRU+FNN model with only pretraining using the LC-PEPTIDE data and with transfer learning on the LC-TEN test data set. In transfer learning, the model is pretrained with the LC-PEPTIDE data and retrained with the LC-TEN training data. (a) The prediction accuracy is R = 0.974 and MAE = 0.0279 for the model with only pretraining. (b) The prediction accuracy is R=0.978 and MAE=0.0271 for the model with transfer learning.

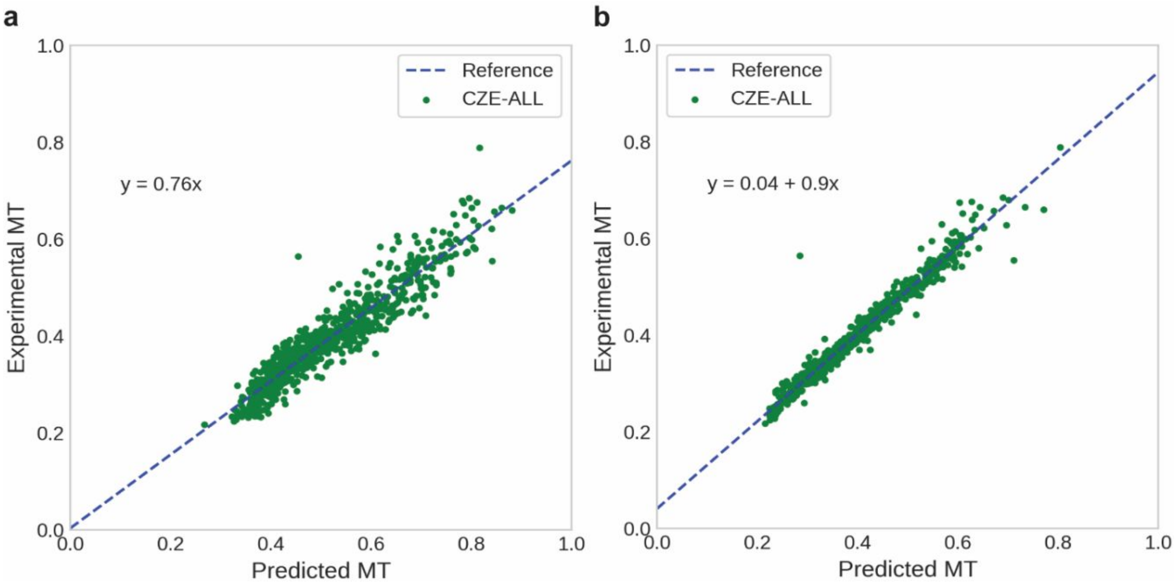

Figure S8. Comparison of MT prediction accuracy of the GRU+FNN model with only pretraining using the CZE-PEPTIDE data and with transfer learning on the CZE-ALL test data set. In transfer learning, the model is pretrained with the CZE-PEPTIDE data and retrained with the CZE-ALL training data. (a) The prediction accuracy is R = 0.943 and MAE = 0.0237 for the model with only pretraining. (b) The prediction accuracy is R=0.982 and MAE=0.0104 for the model with transfer learning.

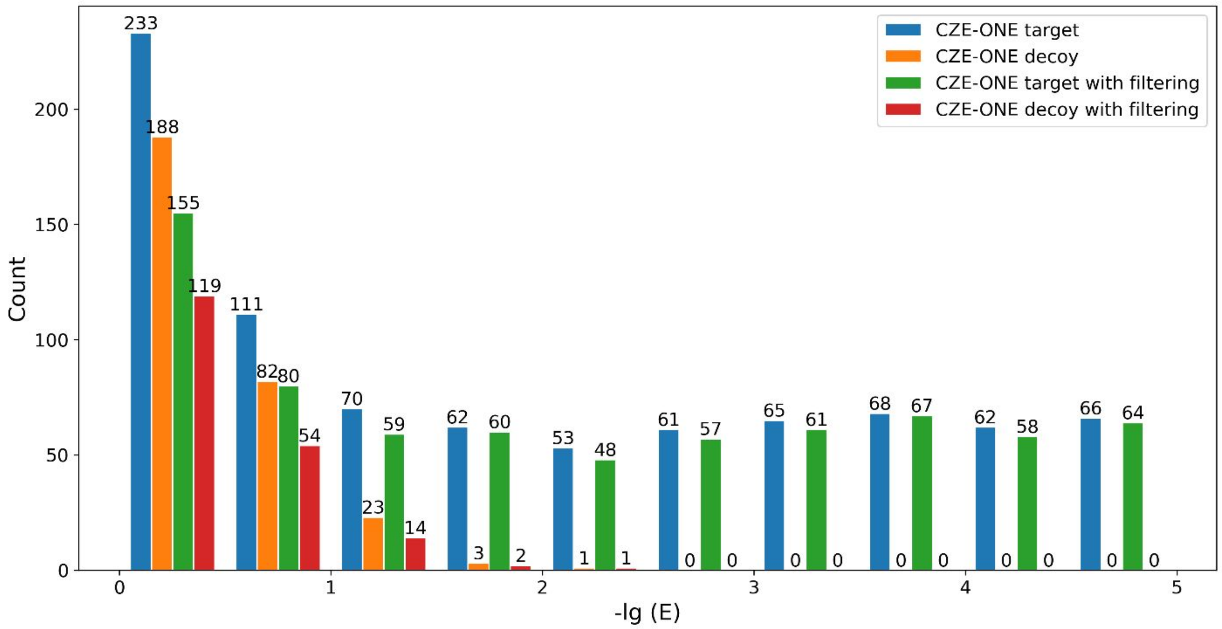

Figure S9. Filtering proteoform identifications using the differences between experimental and theoretical MTs reported by the GRU+FNN model. Target and decoy proteoforms identified from the CZE-ONE data with an *E*-value < 1 are filtered with a cutoff value of 0.1 for experimental and theoretical MT differences. The numbers of target and decoy proteoforms are plotted against their *E*-values with logarithm transformation.

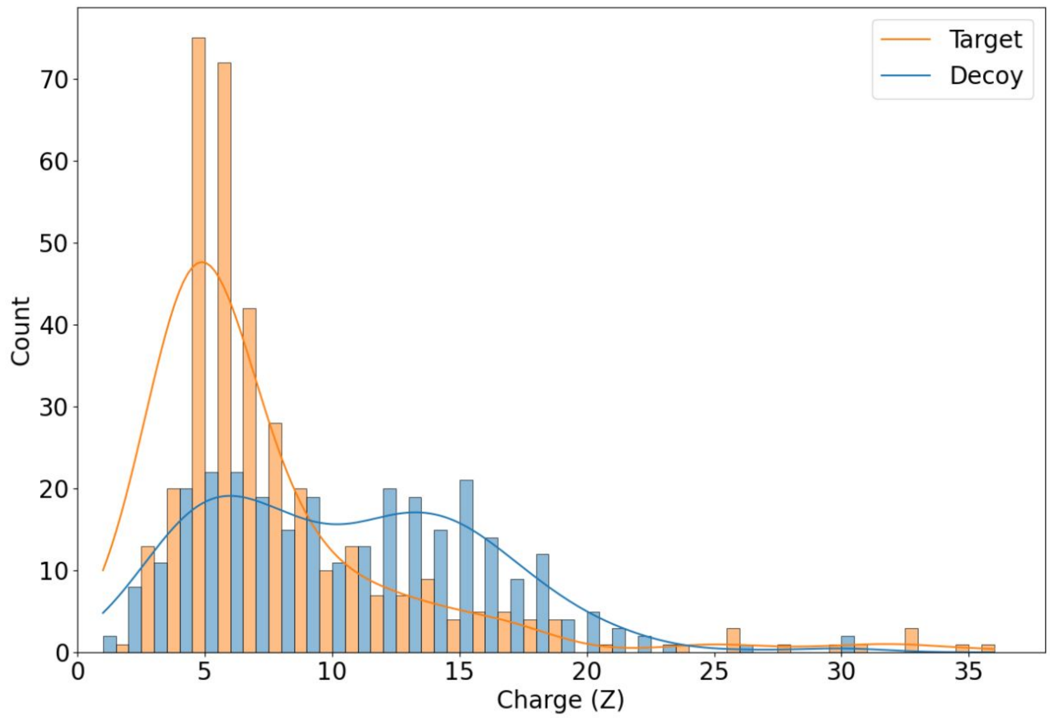

Figure S10. The charge distributions of target and decoy proteoforms identified from the CZE-ONE data set.

References

(1) Ma, C.; Ren, Y.; Yang, J.; Ren, Z.; Yang, H.; Liu, S. Improved peptide retention time prediction in liquid chromatography through deep learning. *Anal. Chem.* **2018**, *90* (18), 10881-10888.
(2) Bouwmeester, R.; Gabriels, R.; Hulstaert, N.; Martens, L.; Degroeve, S. DeepLC can predict retention times for peptides that carry as-yet unseen modifications. *Nat. Methods* **2021**, *18* (11), 1363-1369.
(3) Gessulat, S.; Schmidt, T.; Zolg, D. P.; Samaras, P.; Schnatbaum, K.; Zerweck, J.; Knaute, T.; Rechenberger, J.; Delanghe, B.; Huhmer, A. Prosit: proteome-wide prediction of peptide tandem mass spectra by deep learning. *Nat. Methods* **2019**, *16* (6), 509-518.
(4) Tiwary, S.; Levy, R.; Gutenbrunner, P.; Soto, F. S.; Palaniappan, K. K.; Deming, L.; Berndl, M.; Brant, A.; Cimermancic, P.; Cox, J. High-quality MS/MS spectrum prediction for data-dependent and data-independent acquisition data analysis. *Nat. Methods* **2019**, *16* (6), 519-525.
(5) Yang, Y.; Liu, X.; Shen, C.; Lin, Y.; Yang, P.; Qiao, L. In silico spectral libraries by deep learning facilitate data-independent acquisition proteomics. *Nat. Communications* **2020**, *11* (1), 1-11.
(6) Wen, B.; Li, K.; Zhang, Y.; Zhang, B. Cancer neoantigen prioritization through sensitive and reliable proteogenomics analysis. *Nat. Communications* **2020**, *11* (1), 1-14.
